# Supplementary material for: Docking sites inside Cas9 for adenine base editing diversification and RNA off-target elimination
Source: Nat Commun. 2020 Nov 17;11:5827. doi: 10.1038/s41467-020-19730-9 (PMC7673026; doi:10.1038/s41467-020-19730-9)
Supplement: Supplementary file 1 — Supplementary Information [file 41467_2020_19730_MOESM1_ESM.pdf]

Docking sites inside Cas9 for adenine base editing diversification and RNA off-target elimination

Li et al.

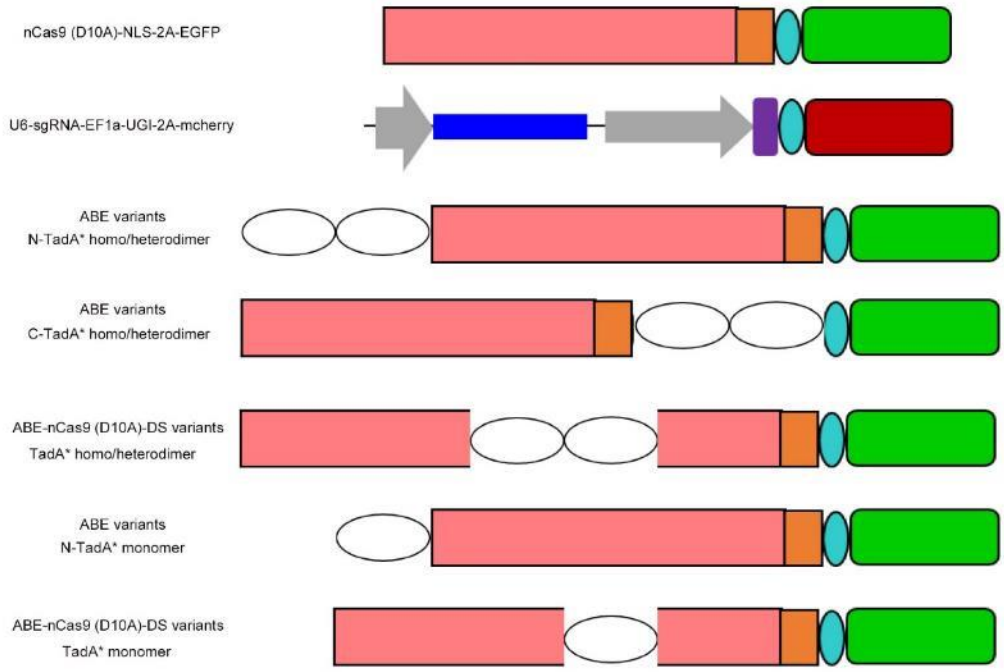

Supplementary Figure 1 Schematic representations of ABE variants

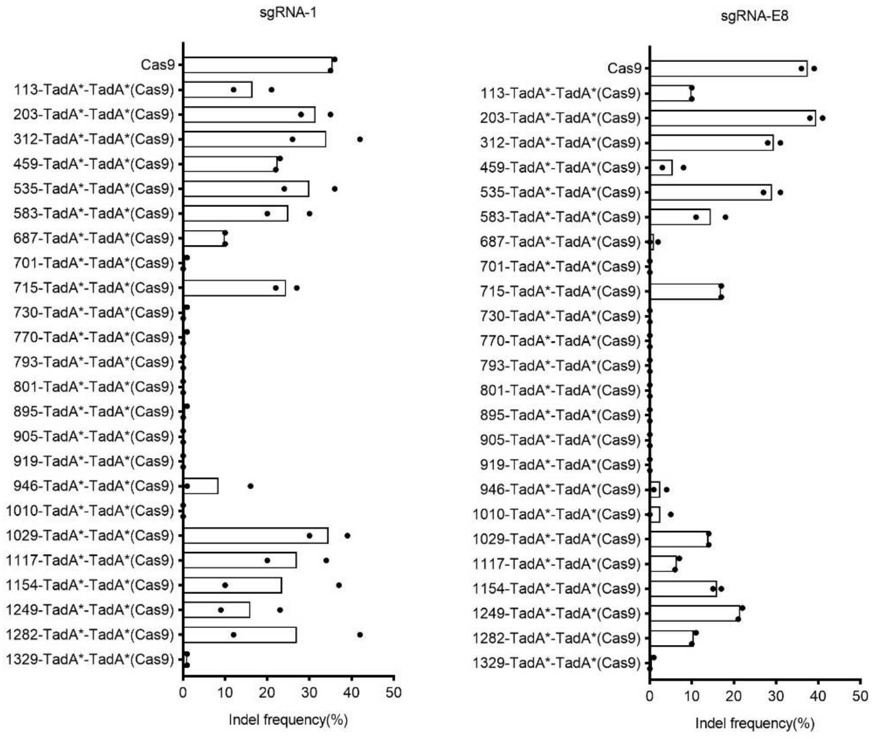

Supplementary Figure 2 DNA cleavage activities of ABE (TadA*)-SpCas9-DS variants.

ABE (TadA*)-SpCas9-DS variants was generated by inserting TadA*-TadA* dimer inside wild-type SpCas9. DNA cleavage activities of ABE (TadA*)-SpCas9-DS variants against sgRNA-1 and sgRNA-E8 were evaluated and quantified with ICE analysis. Data here are represented as mean for two independent experiments.

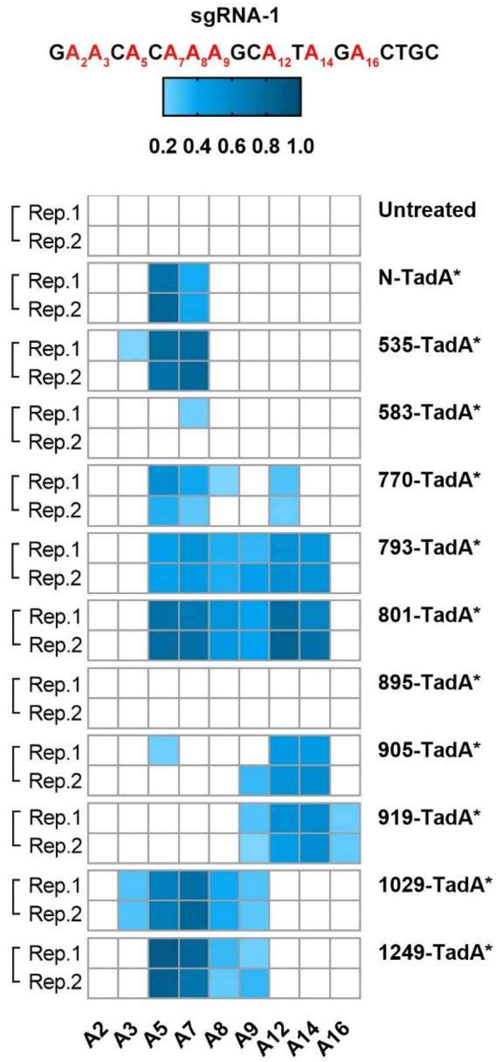

Supplementary Figure 3 Base editing activities of functional ABE (TadA*)-nSpCas9-DS variants.

Base editing activities of ABE (TadA*)-nSpCas9-DS variants against sgRNA-1. A-G conversion frequencies at every adenine nucleotide in 20bp protospacer were quantified with EditR and showed in heat map. Adenines in the sgRNA-1 were labeled as red. Two independent experiments were performed and shown in the heat map.

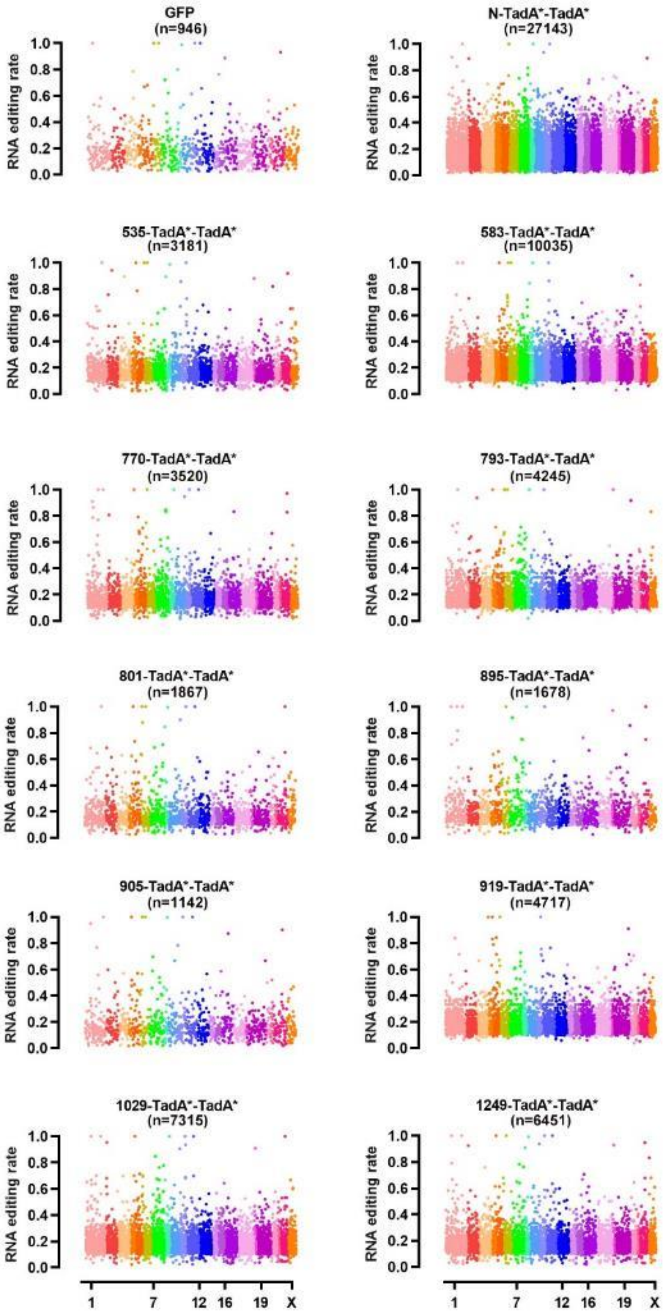

Supplementary Figure 4 RNA off-target activities of functional ABE (TadA*-TadA*)-nSpCas9-DS variants and N-terminal counterpart.

Representative distributions of edited adenine nucleotides on different chromosomes (shown in different colors) for selected ABE (TadA*-TadA*)-nSpCas9-DS variants and N-terminal counterpart. The n represents the number of edited adenine nucleotides.

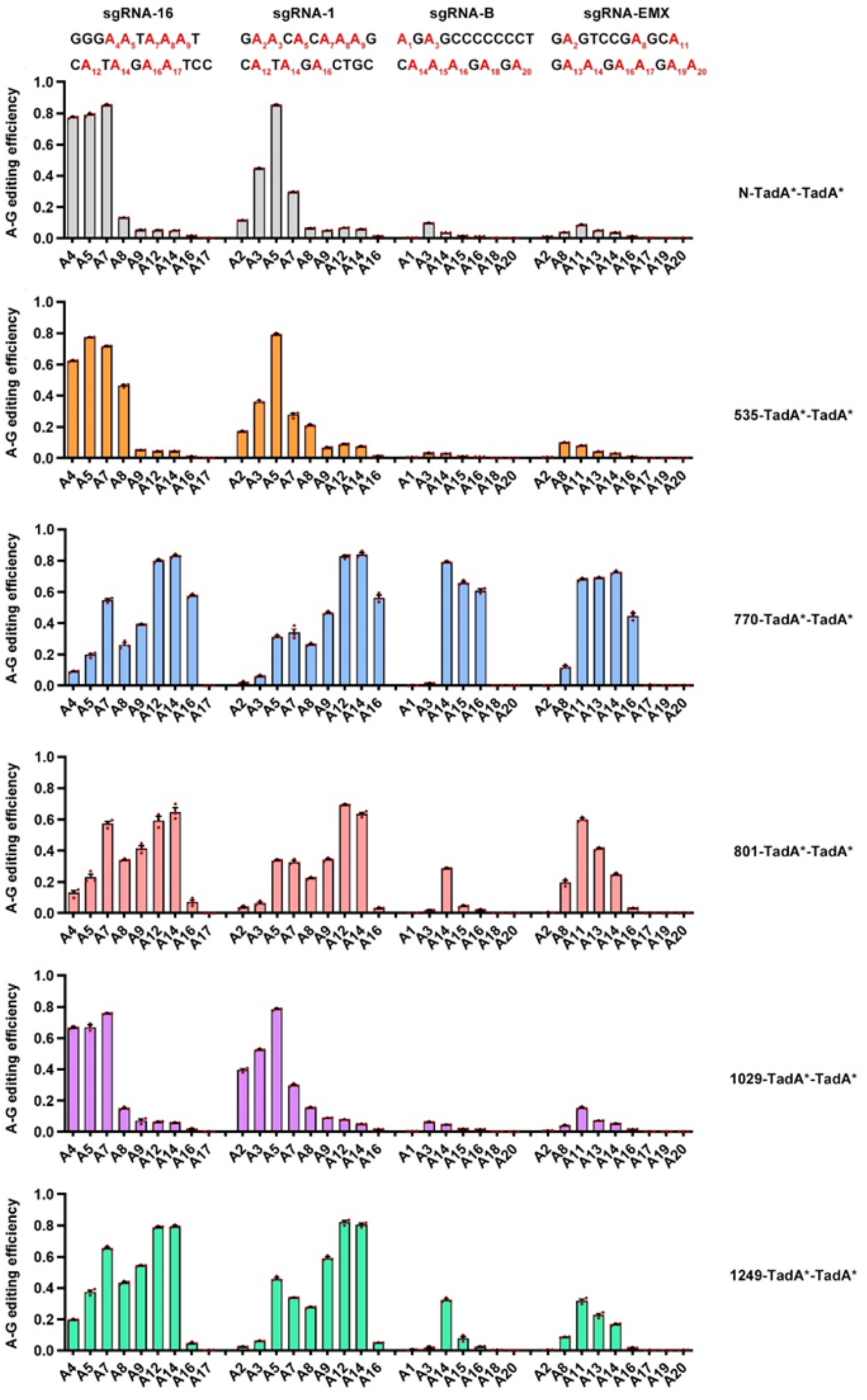

Supplementary Figure 5 Base editing activities of selected ABE (TadA*-TadA*)-nSpCas9-DS variants and N-terminal counterpart across four sgRNA sites.

(Adenines in the sgRNAs were labeled as red. Data here are represented as mean with S.E.M. from three biologically independent experiments.)

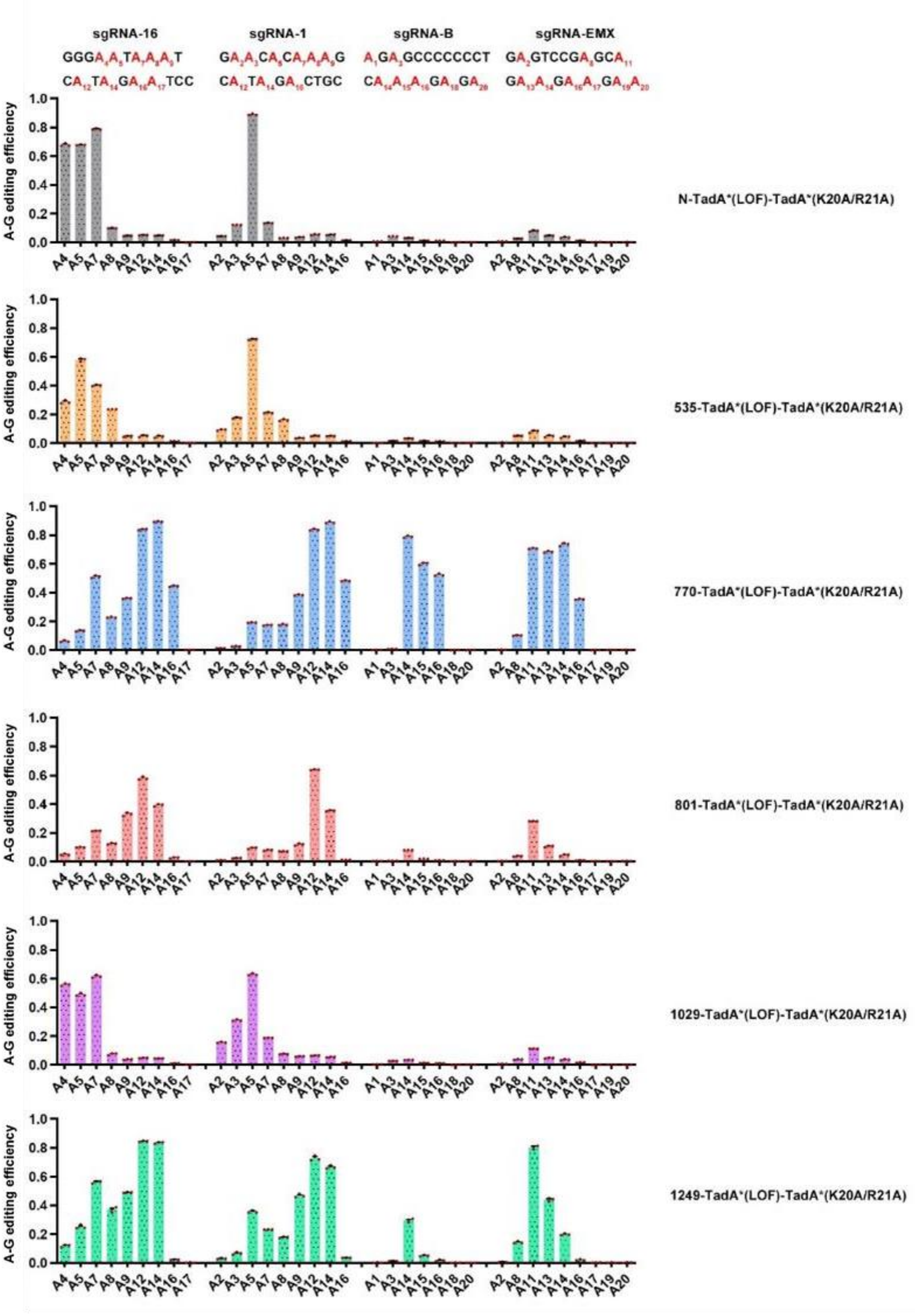

Supplementary Figure 6 Base editing activities of selected ABE (TadA*(LOF)-TadA*(K20A/R20A))-nSpCas9-DS variants and N-terminal counterpart across four sgRNA sites.

(Adenines in the sgRNAs were labeled as red. Data here are represented as mean with S.E.M. from three biologically independent experiments.)

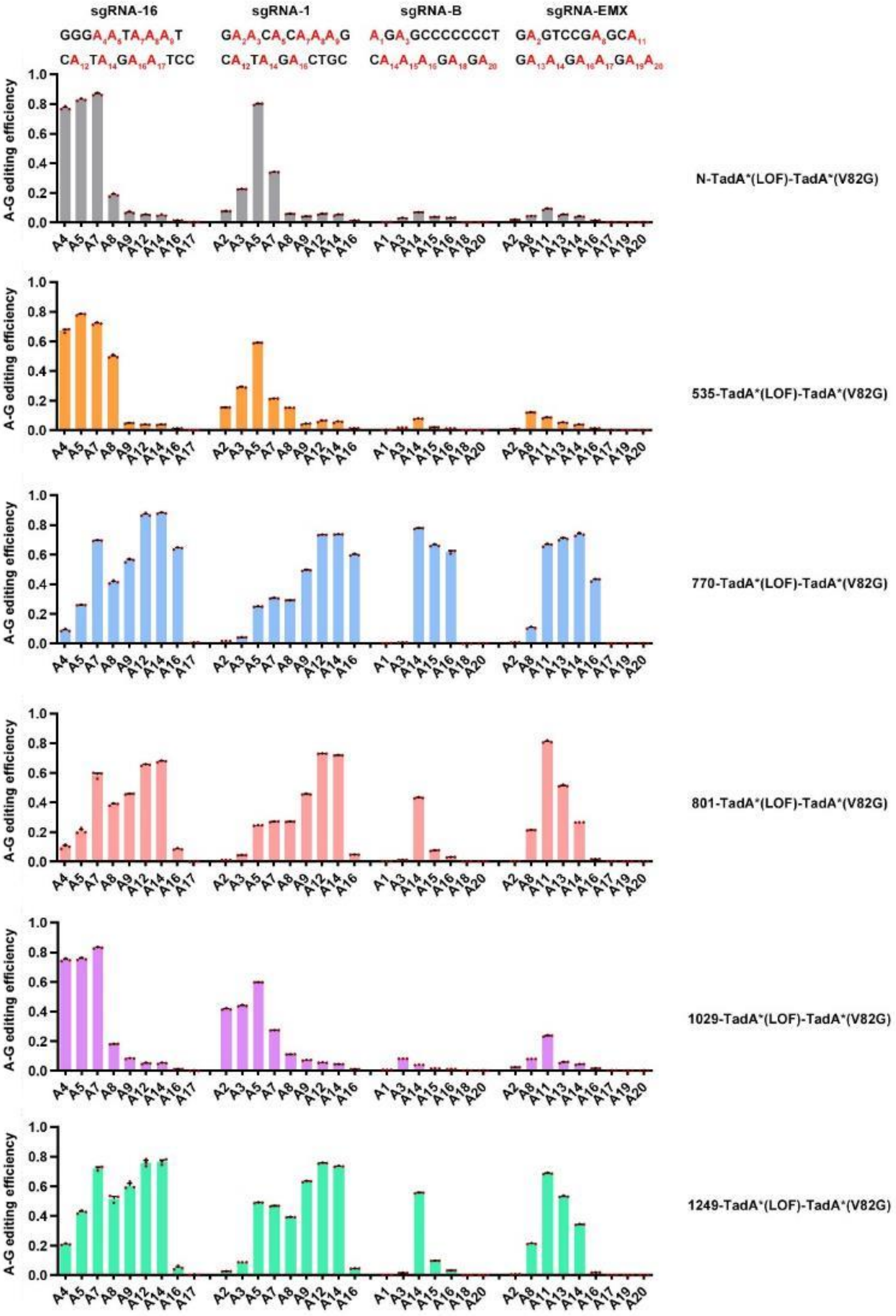

Supplementary Figure 7 Base editing activities of selected ABE (TadA*(LOF)-TadA*(V82G))-nSpCas9-DS variants and N-terminal counterpart across four sgRNA sites.

(Adenines in the sgRNAs were labeled as red. Data here are represented as mean with S.E.M. from three biologically independent experiments.)

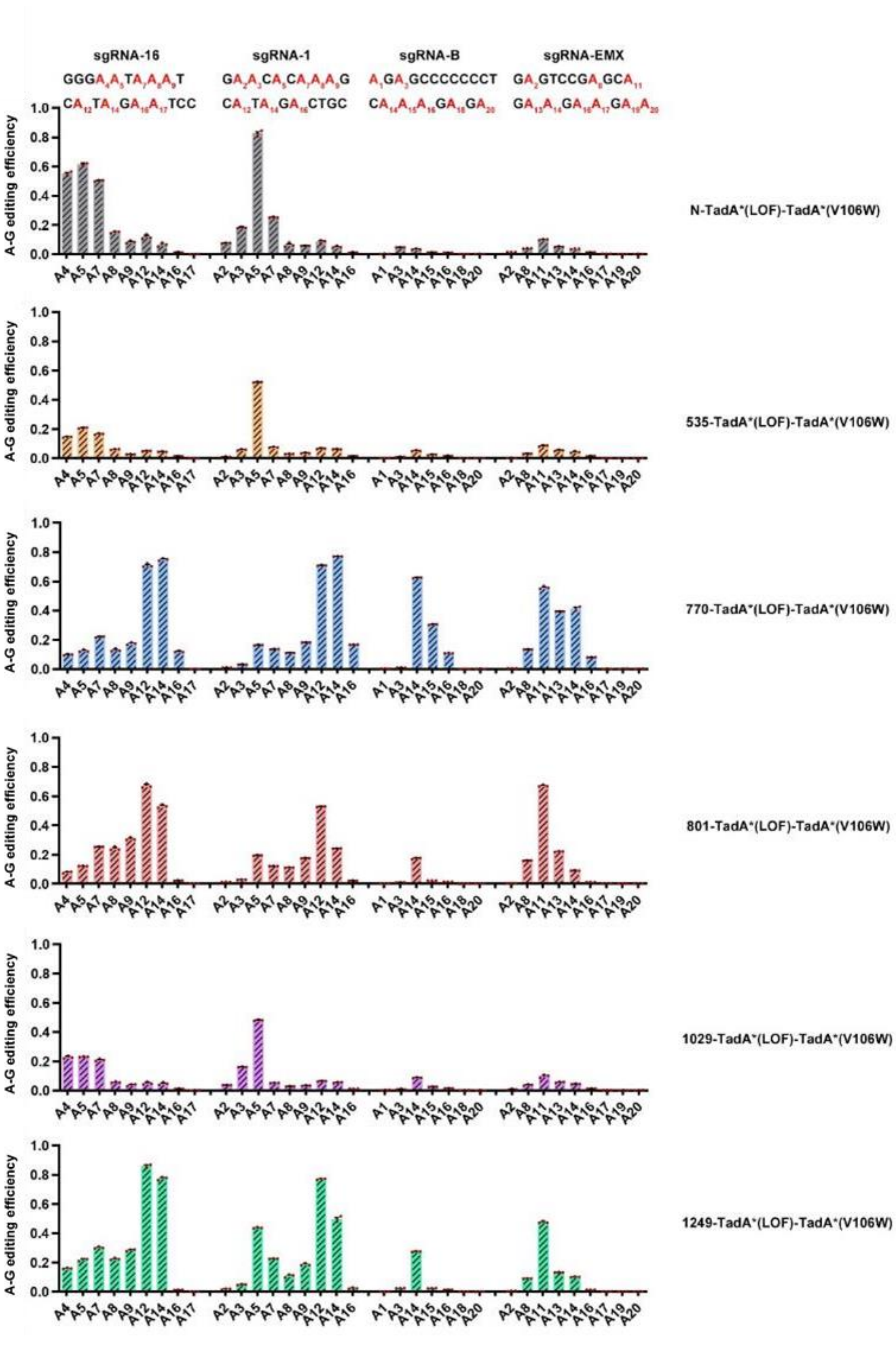

Supplementary Figure 8 Base editing activities of selected ABE (TadA*(LOF)-TadA*(V106W))-nSpCas9-DS variants and N-terminal counterpart across four sgRNA sites.

(Adenines in the sgRNAs were labeled as red. Data here are represented as mean with S.E.M. from three biologically independent experiments.)

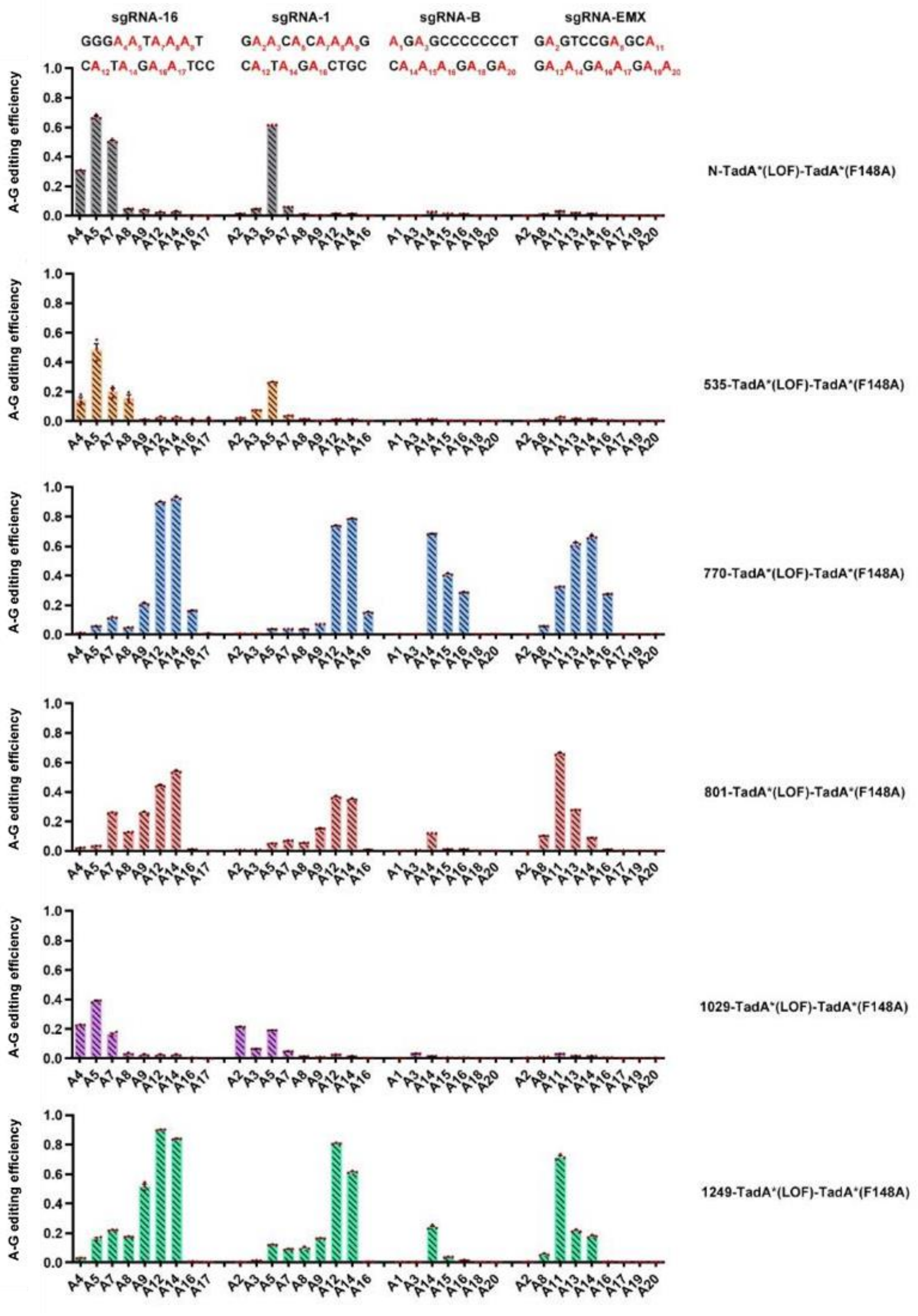

Supplementary Figure 9 Base editing activities of selected ABE (TadA*(LOF)-TadA*(F148A))-nSpCas9-DS variants and N-terminal counterpart across four sgRNA sites.

(Adenines in the sgRNAs were labeled as red. Data here are represented as mean with S.E.M. from three biologically independent experiments.)

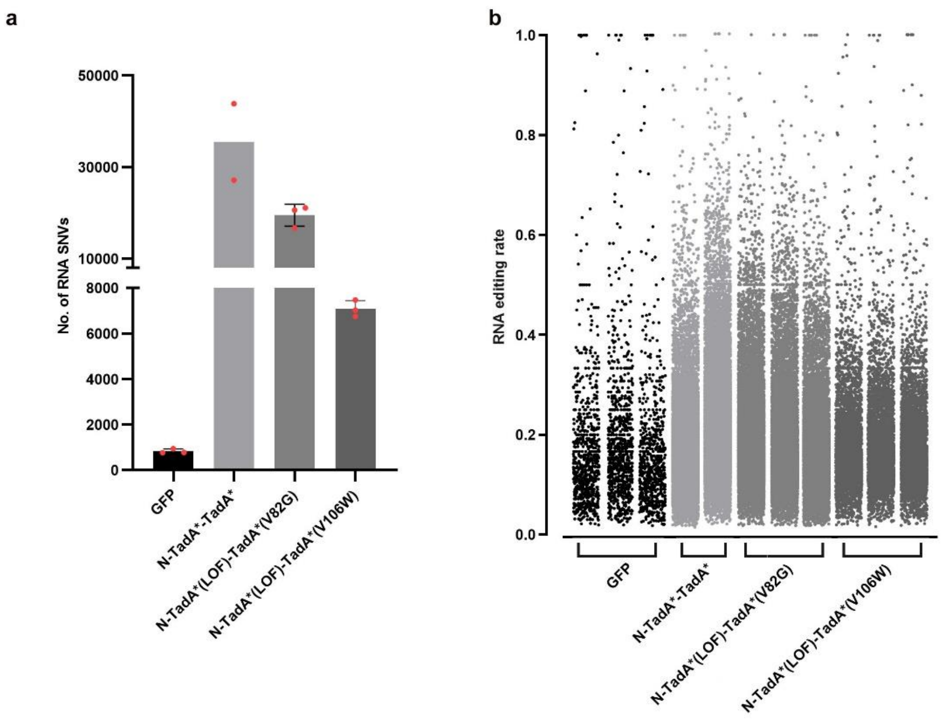

Supplementary Figure 10 RNA off-target activities of N-TadA*-TadA*, N-TadA*(LOF)-TadA*(V82G) and N-TadA*(LOF)-TadA*(V106W).

(a) Transcriptome analysis showing the number of edited adenine nucleotides in HEK293T cells transfected with N-TadA*-TadA*, N-TadA*(LOF)-TadA*(V82G) or N-TadA*(LOF)-TadA*(V106W) and sgRNA-1. (n=3 for all groups and represented as mean with S.E.M., in addition that n=2 biologically independent samples for N-TadA*-TadA* group)

(b) Jitter plots displaying the RNA A-to-I conversion frequencies at transcriptome level in HEK293T cells transfected with N-TadA*-TadA*, N-TadA*(LOF)-TadA*(V82G) or N-TadA*(LOF)-TadA*(V106W) and sgRNA-1.

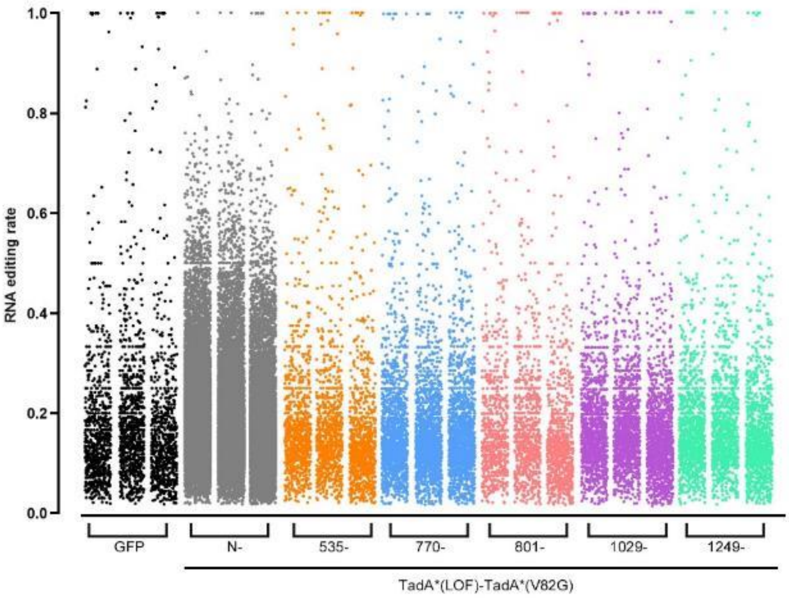

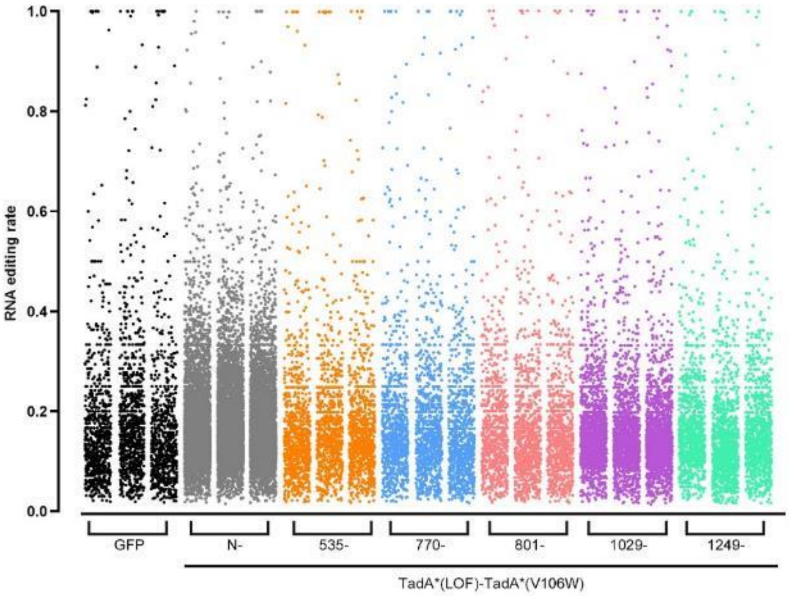

Supplementary Figure 11 RNA off-target activities of selected ABE-nSpCas9-DS variants and N-terminal counterpart in jitter plots

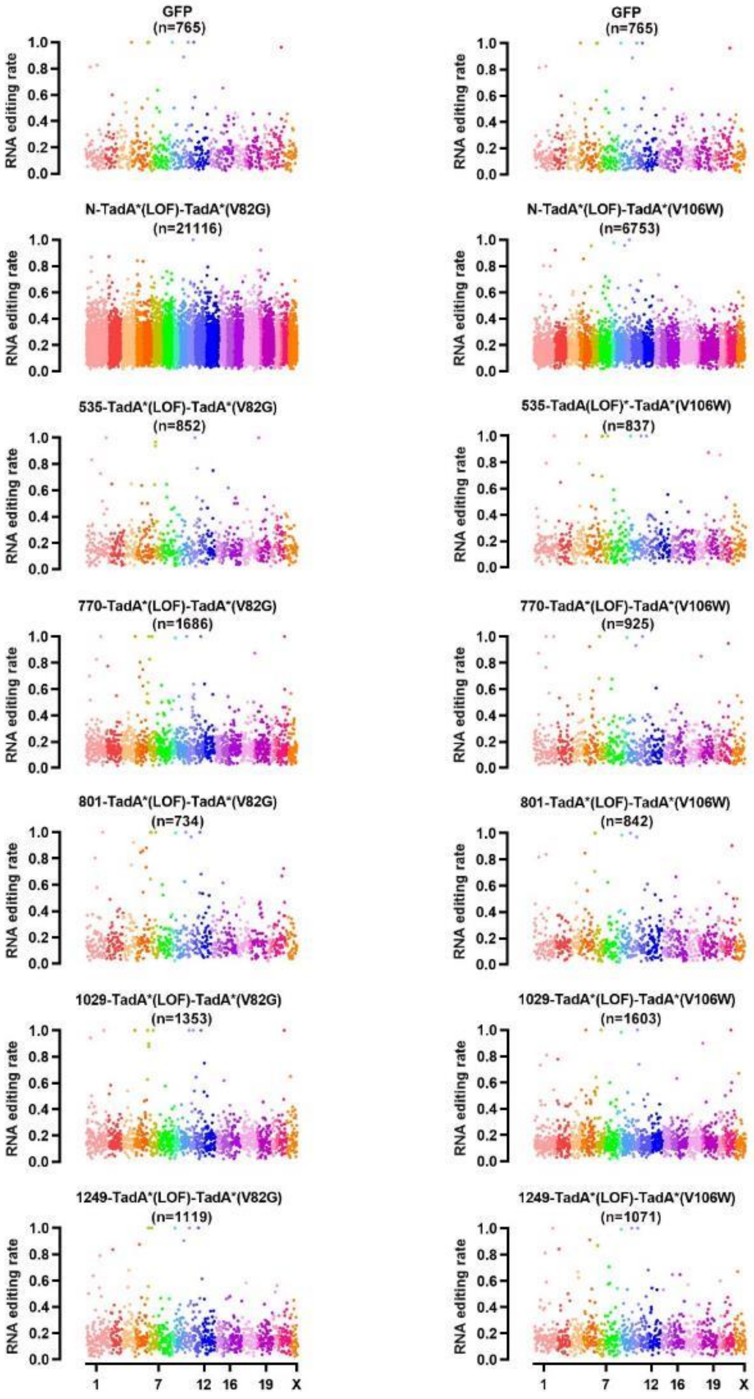

Supplementary Figure 12 Representative distributions of edited adenine nucleotides on different chromosomes for selected ABE variants

Representative distributions of edited adenine nucleotides on different chromosomes for selected ABE variants for selected ABE (TadA*(LOF)-TadA*(V82G))-nSpCas9-DS variants including N-terminal counterpart (left) and selected ABE (TadA*(LOF)-TadA*(V106W))-nSpCas9-DS variants including N-terminal counterpart (right).

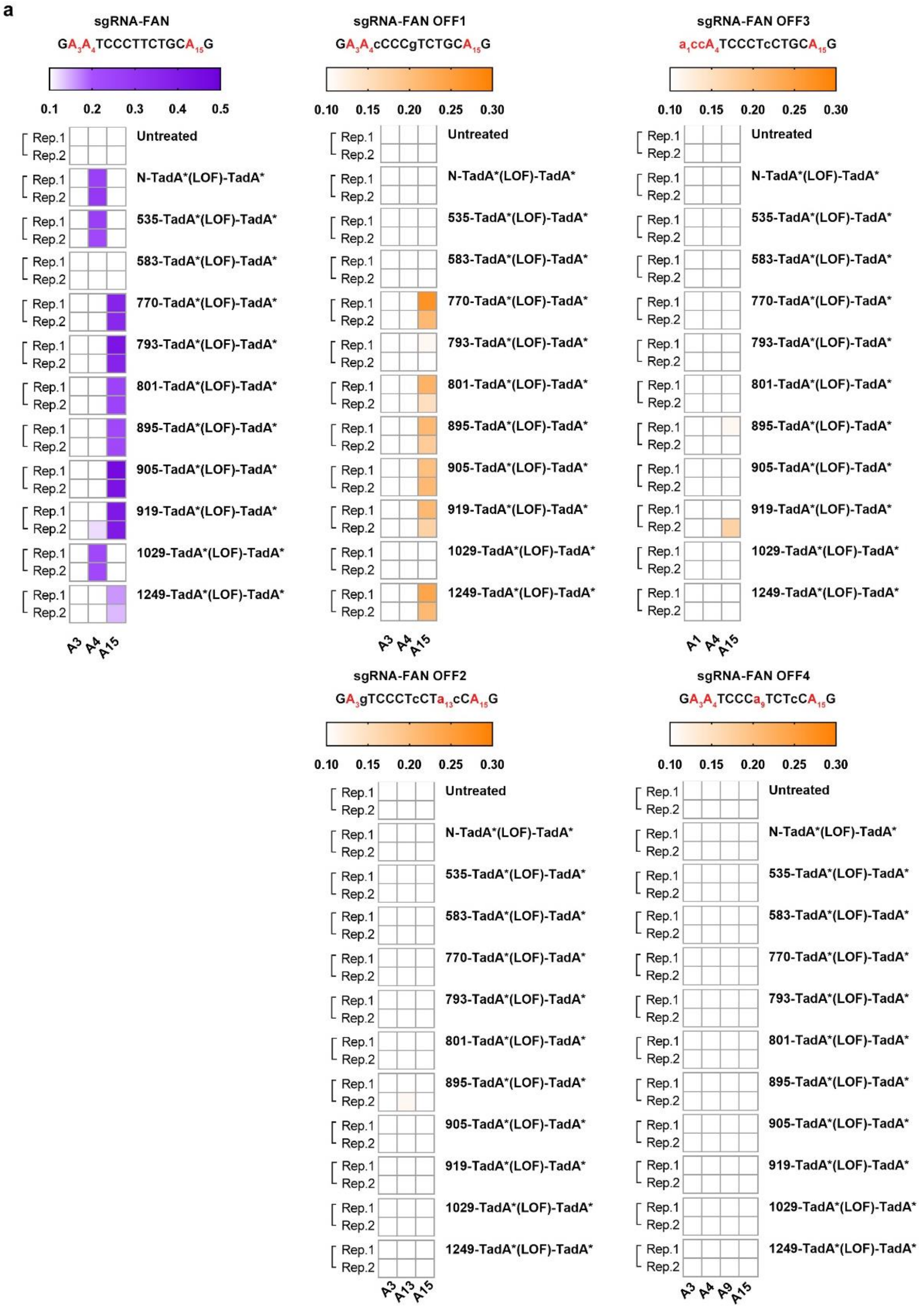

Supplementary Figure 13 DNA off-target activities of functional ABE (TadA* (LOF)-TadA*)-nSpCas9-DS variants at FANCF site in HEK293T cells

Base editing activities of functional ABE (TadA* (LOF)-TadA*)-nSpCas9-DS variants at FANCF site (purple) and four potential off-target sites (orange) were quantified using EditR and shown in heat maps. Adenines in the sgRNAs were

labeled as red. Two independent experiments were performed and shown in the heat map.

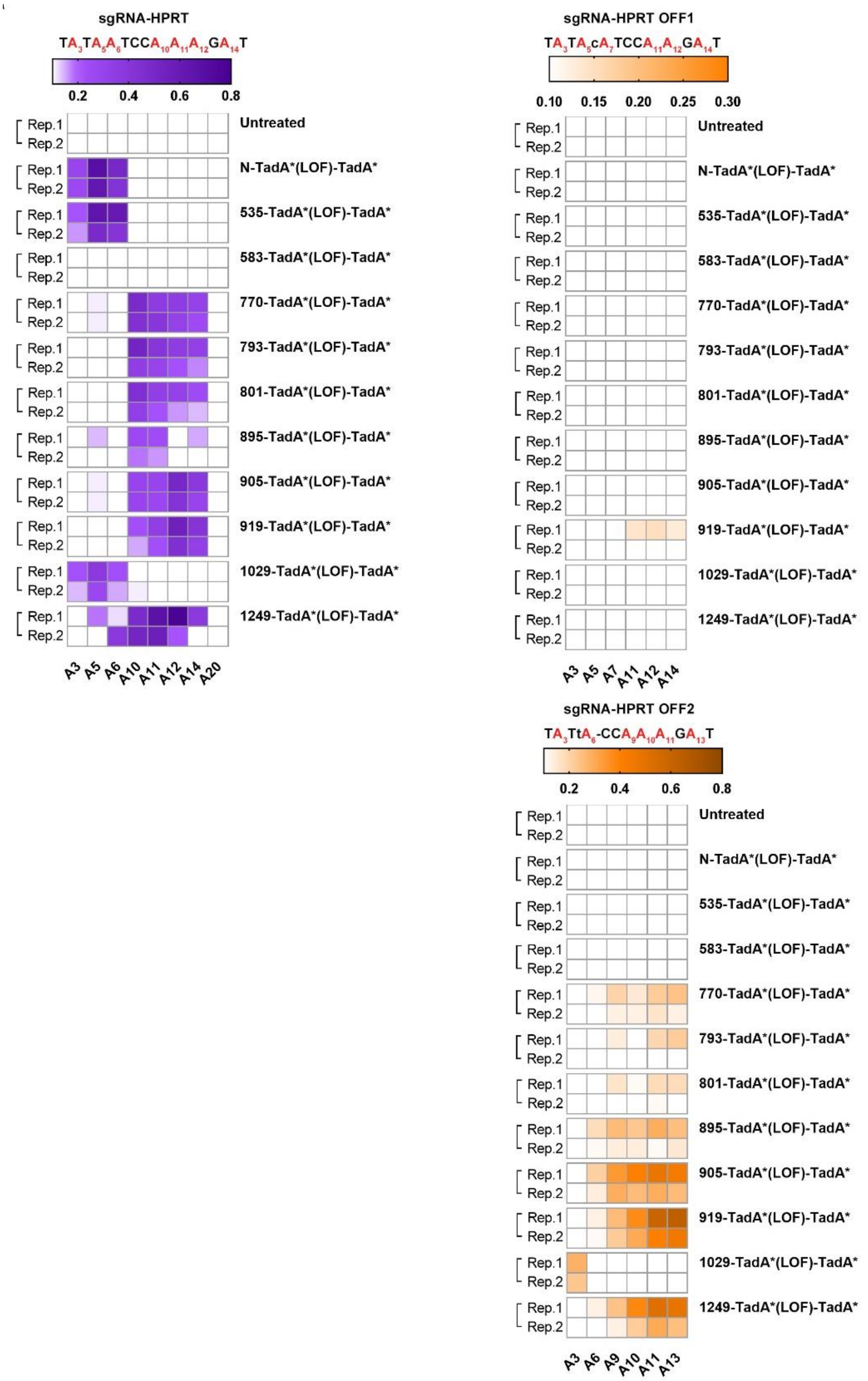

Supplementary Figure 14 DNA off-target activities of functional ABE (TadA* (LOF)-TadA*)-nSpCas9-DS variants at HPRT site in HEK293T cells

Base editing activities of functional ABE (TadA* (LOF)-TadA*)-nSpCas9-DS variants at FANCF site (purple) and the top two potential off-target sites (orange) were quantified using EditR and shown in heat maps. Adenines in the sgRNAs were

labeled as red. Two independent experiments were performed and shown in the heat map.

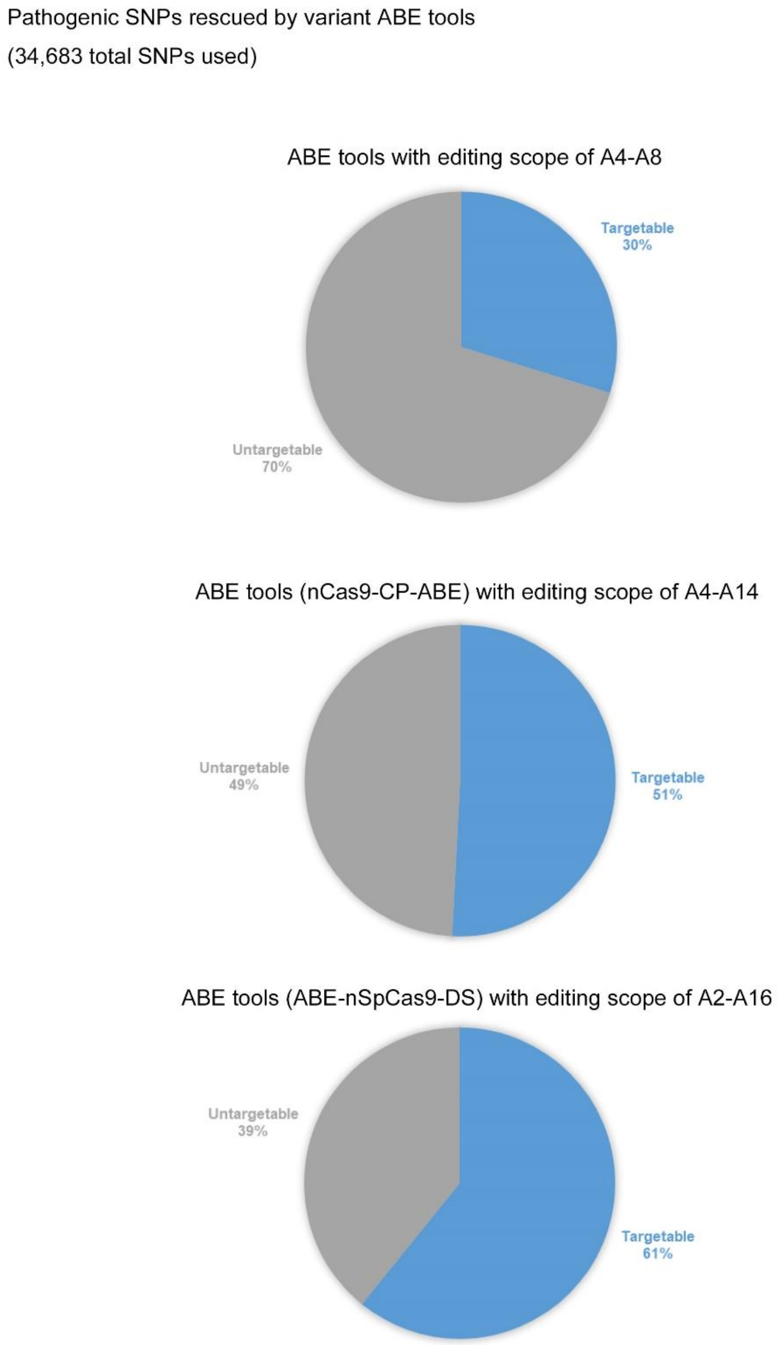

Supplementary Figure 15 The percentage of human pathogenic SNPs correctable by functional ABE-nSpCas9-DS variants

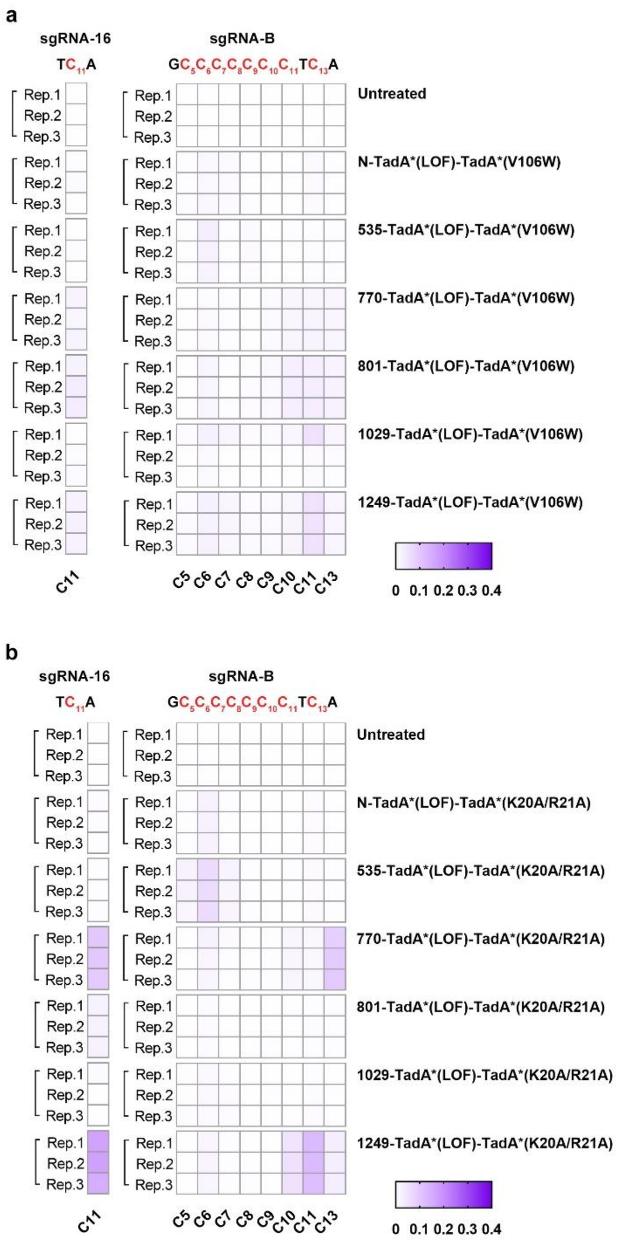

Supplementary Figure 16 Cytosine deamination activities of selected ABE-nSpCas9-DS variants and N-terminal counterpart

Cytosine deamination activities of selected ABE (TadA*(LOF)-TadA*(V106W))-nSpCas9-DSs (a), ABE (TadA*(LOF)-TadA*(K20A/R21A))-nSpCas9-DSs (b) and N-terminal counterpart against sgRNA-B and sgRNA-16. Cytosines in the sgRNAs were

labeled as red. Two independent experiments were performed and shown in the heat map.

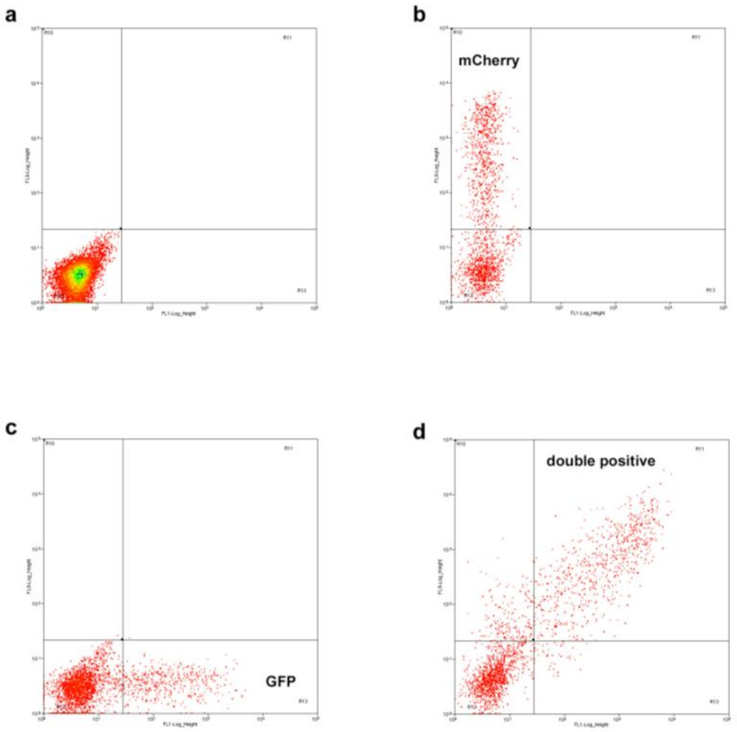

Supplementary Figure 17 Gating strategy for cell sorting

(a) HEK293T cells without transfection were used as negative control and neither mCherry positive nor GFP positive cells were detected.

(b) HEK293T cells transfected with single plasmid expressing both specific sgRNA and mCherry simultaneously. Gating strategy for mCherry-positive cells were labeled as mCherry.

(c) HEK293T cells transfected with single plasmid expressing both base editors and GFP simultaneously. Gating strategy for GFP-positive cells were labeled as GFP.

(d) HEK293T cells transfected with two plasmids. One expressed sgRNA and mCherry while another expressed base editors and GFP. Gating strategy for double positive cells were labeled as double positive. In this study, only double positive cells were collected for base editing analysis and RNA off-target analysis.

Supplementary Table 1: Summarized sgRNA information including sgRNA sequences, locations in DNA strand and positions in PCR products.

sgRNA name	sgRNA sequence
SgRNA-B	AGAGCCCCCCCTCAAAGAGA
SgRNA-16	GGGAATAAATCATAGAATCC
SgRNA-1	GAACACAAAGCATAGACTGC
SgRNA-EMX	GAGTCCGAGCAGAAGAAGAA
SgRNA-E8	GGCTGAGCTGAGAGCCTGAT
SgRNA-FAN	GGTGCTGCAGAAGGGATTCC
SgRNA-HPRT	GTATAATCCAAAGATGGTCA
SgRNA-HDAC	GGAGAAGACAGACAGAGGGC
SgRNA-SOD	GCCAGACTTAAATCACAGAT
SgRNA-PT14	TCCAAACCCATATATACAGC
SgRNA-PT13	CCAAACCCATATATACAGCA
SgRNA-PCBP	GTCGATAAGAAATGTAAGAG
SgRNA-MSSMA	ACCCACGGCGGGGATCAGGG
SgRNA-HBD	GAACACAATGCCTACTTCAA
SgRNA-H42	TGCTGTGTGACTACAGTGG
SgRNA- FANMC	GTAACGAGCTGCATCCCCGA
SgRNA-EMX2	GTATTCACCTGAAAGTGTGC
SgRNA-CHD71	GCCATAAATCAAAACTCAC
SgRNA-824	GATGTAGGGCTAGAGGGGTG
SgRNA-182	CAGGCTCCCAGGTAATGCTT
SgRNA-162	AGAAATTTTCTCTATCCACC
SgRNA-122	TAGAATAGTAGTAAAAAAAC
SgRNA-FAAAC	GGCGGCTGCACAACCAGTGG
SgRNA-RNF2	GTCATCTTAGTCATTACCTG
SgRNA-830	GTTGGAGCGGGGAGAAGGCC
SgRNA-103	GGGAGGTGGAGGATAATGTG
SgRNA-163	ACCCTTTTTGAATACCTGAT

Supplementary Discussion

It has been demonstrated that SpCas9 protein could be engineered in many different ways while maintaining its RNA-guided double-stranded DNA (dsDNA) binding and cleavage ability. For example, Cas9 could be split into two fragments to establish a functional split-Cas9 system for inducible genome modification[1-3]. Additionally, hotspots inside Cas9 tolerating mouse alpha1-syntrophin (PDZ) domain insertions have been identified[4] and Cas9 could be rearranged via protein circular permutation strategy[5]. We speculate that the locations of split sites, functional insertional hotspots and hotspots of functional Cas9 circular permutants represent the potential regions tolerating additional domain insertions. In consideration of reported functional regions, we initially selected potential docking sites (DSs) around regions suitable for at least 2 out of 3 Cas9 engineering strategies including split-Cas9 construction, PDZ domain insertion and Cas9 circular permutation. These regions include DS^{113}, DS^{203}, DS^{312}, DS^{459}, DS^{535}, DS^{687}, DS^{715}, DS^{801}, DS^{946}, DS^{1010}, DS^{1029}, DS^{1117}, DS^{1154}, DS^{1249} and DS^{1282}. As site 573-574 and site 713-714 have been used widely for functional split-Cas9 system construction, we further selected DS^{583}, DS^{701} and DS^{730} to evaluate their tolerating ability for adenosine deaminase insertions. Additionally, as HNH domain is intrinsically flexible inside Cas9 and close to ssDNA loop[6], we also selected DS^{770}, DS^{793}, DS^{895}, DS^{905} and DS^{919} to assess their tolerating ability for adenosine deaminase insertions. As DS^{1329} is near the PAM region, we wondered whether adenosine deaminase in this region could achieve A-G conversion around A17-A20, which are untargetable with existing ABE tools. The final 24 potential docking sites (DSs) are distributed across different domains of Cas9 and mainly in discrete and flexible regions. Thus, these 24 DSs could provide a relatively systematic assessment for the impact of adenosine deaminase insertions in SpCas9. However, as SpCas9 contains 1368 amino acids, a more comprehensive analysis for other regions is still needed in the future.

1. Zetsche, B., Volz, S.E. & Zhang, F. A split-Cas9 architecture for inducible genome editing and transcription modulation. *Nat Biotechnol* **33**, 139-42 (2015).
2. Nihongaki, Y., Kawano, F., Nakajima, T. & Sato, M. Photoactivatable CRISPR-Cas9 for optogenetic genome editing. *Nat Biotechnol* **33**, 755-60 (2015).
3. Truong, D.J. et al. Development of an intein-mediated split-Cas9 system for gene therapy. *Nucleic Acids Res* **43**, 6450-8 (2015).
4. Oakes, B.L. et al. Profiling of engineering hotspots identifies an allosteric CRISPR-Cas9 switch. *Nat Biotechnol* **34**, 646-51 (2016).
5. Oakes, B.L. et al. CRISPR-Cas9 Circular Permutants as Programmable Scaffolds for Genome Modification. *Cell* **176**, 254-267 e16 (2019).
6. Nishimasu, H. et al. Crystal structure of Cas9 in complex with guide RNA and target DNA. *Cell* **156**, 935-49 (2014).
