# Supplementary material for: Docking sites inside Cas9 for adenine base editing diversification and RNA off-target elimination
Source: Nat Commun. 2020 Nov 17;11:5827. doi: 10.1038/s41467-020-19730-9 (PMC7673026; doi:10.1038/s41467-020-19730-9)
Supplement: Supplementary file 8 — Description of Additional Supplementary Files [file 41467_2020_19730_MOESM8_ESM.pdf]

Title: Supplementary Data 1
Descriptions: Primers used for mutagenesis

Title: Supplementary Data 2
Descriptions: Sanger Sequencing data summary

Title: Supplementary Data 3
Descriptions: Deep-Seq data summary

Title: Supplementary Data 4
Descriptions: Primers used for PCR amplifications
